# The prognostic validity of the formative for the summative MEQ (Modified Essay Questions) 

**DOI:** 10.3205/zma001495

**Published:** 2021-09-15

**Authors:** Oliver Büssing, Jan P. Ehlers, Michaela Zupanic

**Affiliations:** 1 Klinikum Westfalen, Hellmig Hospital Kamen, Medical Clinic I - Clinic for Angiology, Cardiology and Intensive Care Medicine, Kamen, Germany; 2 Witten/Herdecke University, Faculty of Health, Chair Didactics and Educational Research in Health Care, Witten, Germany; 3 Witten/Herdecke University, Faculty of Health, Interprofessional and Collaborative Didactics in Medical and Health Professions, Witten, Germany

**Keywords:** Modified Essay Questions (MEQ), formative testing, summative testing, predictors of academic success, constructive alignment

## Abstract

**Objective:** The purpose of formative examinations is that students and lecturers receive an early feedback regarding the success of learning behavior and teaching methods. These also serve as practice for later summative exams. The aim of this paper is to investigate to what extent the result of the formative MEQ* at the end of the first semester at Witten/Herdecke University (UW/H) in the study program human medicine can be used as a predictor for the summative MEQ-1 at the end of the second semester which is part of the equivalence examination replacing the state examination.

**Methodology: **The predictive value of the score achieved in the MEQ* on the MEQ-1 score, as well as the potential influence of the variables gender, age, high school graduation grade (German Abiturnote), professional background, and self-efficacy expectancy, was determined for students of human medicine.

**Results: **Data from two cohorts of UW/H with a total of 88 students were included. Scores on the formative MEQ* correlate with those on the summative MEQ-1 in both cohorts. In regression analyses, only the score on the MEQ* proves to be a significant predictor of performance on the MEQ-1 (40.5% variance explanation). Particularly significant predictors are the scores in the subjects anatomy and clinical reasoning. Vocational training or pre-study only appear to contribute to higher scores in the MEQ* after the first semester, but have no further significance in predicting scores in the MEQ-1.

**Conclusion:** The MEQ* was confirmed to be a good predictor of the MEQ-1. Thus, it serves as a formative exam to inform students about their current state of knowledge with regard to the summative exam MEQ-1, so that they can adequately adapt their learning strategies in the course of the second semester.

## Zusammenfassung

**Zielsetzung:** Damit Studierende und Dozierende frühzeitig ein Feedback bezüglich des Erfolgs von Lernverhalten und Lehrmethoden bekommen, werden formative Prüfungen geschrieben. Diese dienen auch als Übung für spätere summative Prüfungen. Ziel dieser Arbeit ist die Untersuchung, in wie weit das Ergebnis des formativen MEQ* am Ende des 1. Semesters an der Universität Witten/Herdecke (UW/H) im Studiengang Humanmedizin als Prädiktor für den summativen MEQ-1 am Ende des 2. Semesters, der Teil der Staatsexamens ersetzenden Äquivalenzprüfung ist, dienen kann.

**Methodik: **Der prädiktive Wert der Punktzahl aus dem MEQ* auf die Punktzahl des MEQ-1 sowie der mögliche Einfluss der Variablen Geschlecht, Alter, Abiturnote, berufliches Vorwissen und Selbstwirksamkeitserwartung wurde für die Studierenden der Humanmedizin ermittelt.

**Ergebnisse: **Einbezogen wurden Daten von zwei Jahrgängen der UW/H mit insgesamt 88 Studierenden. Die Punktzahlen im formativen MEQ* korrelieren für beide Jahrgänge mit denen des summativen MEQ-1. In Regressionsanalysen erweist sich lediglich die Punktzahl im MEQ* als signifikanter Prädiktor der Leistung im MEQ-1 (40.5% Varianzerklärung). Besonders bedeutsame Prädiktoren sind die Ergebnisse in den Fächern Anatomie und Klinisches Denken. Eine Berufsausbildung oder ein Vorstudium scheinen nur zu höheren Punktzahlen im MEQ* nach dem ersten Semester beizutragen, haben aber für die Prädiktion der Punktzahl im MEQ-1 keine weitere Bedeutung.

**Schlussfolgerung: **Der MEQ* konnte als guter Prädiktor für den MEQ-1 bestätigt werden. Somit kommt dieser seiner Funktion als formative Prüfung nach, die Studierenden über ihren aktuellen Wissensstand im Hinblick auf die summative Prüfung MEQ-1 zu informieren, damit diese ihre Lernstrategien im Verlauf des 2. Semesters adäquat anpassen können.

## Introduction

Since a university course of study is associated with special challenges, it is of particular interest to students and lecturers to find out at an early stage whether learning behavior and teaching methods are goal-oriented. According to the constructive alignment concept, teaching content, learning outcomes and examination formats must be coherently related to each other [1], [2]. Besides the quality criteria of objectivity and reliability, the basic prerequisite for a valid examination is therefore testing of the content that has previously been defined in the learning outcomes [3]. Through examinations during the course of study, students receive regular feedback on the current state of their knowledge. In this context, formative testing aims to monitor and test a (learning) program that is still in progress [4], [5]. The individual feedback provides valuable guidance for the students' learning plan and the opportunity to reflect on their learning strategies. Formative testing can thus support intrinsic motivation [6]. Summative tests, on the other hand, are used to assess competence, evaluate an already completed (learning) program, and entitle students to more advanced educational segments [4]. However, they may not provide sufficient feedback to specifically support student’s learning, but can influence and direct it, in the sense of extrinsic learning motivation. This is often socially reinforced by fellow students [7]. 

An overview of the examinations used in medical education can be found in Epstein [8] and Schuwirth and van der Vleuten [9] and for the DACH countries in Thiessen et al. [10]. Written exams can be roughly divided into two groups: Closed-ended response, such as multiple choice questions (MCQ), and open-ended response, such as free-response essays [11]. As a synthesis of these two basic examination formats, the first MEQ (Modified Essay Questions) was developed as an examination for general practitioners in the UK in 1971 [12], [13]. MEQ (Modified Essay Questions) is intended to combine the reliability and objectivity of an MCQ examination with the validity of an essay. The tasks in the MEQ consist of a clinical case example, which is first described using a brief outline with a reason for treatment. The following questions, which build upon each other, have to be answered using short free texts [11]. This shall provide both a high level of cognitive challenge and a largely standardized correction of the questions. Free-text tasks are well suited for testing clinical reasoning, but at the same time require a relatively high effort in the formulation of the answer horizons and score distribution and in the correction [14], [15]. The content of the tasks relates to the medical activities

1. history taking, diagnosis, and therapy,
2. differential diagnostic thinking and problem-solving strategies, and
3. holistic thinking and judgment [16].

In order to adequately master the clinical decision-making process simulated in the case study of a real situation, students must actively reproduce or apply their knowledge [17]. An MEQ can test knowledge with regard to the modified competence levels according to Bloom [18] not only at level I (factual knowledge), but also at levels II (conceptual knowledge) and III (procedural knowledge).

At Witten/Herdecke University (UW/H), problem-based learning (POL) was introduced in 1992 in the then reformed course of study in human medicine and established in 2000 as an interdisciplinary concept in the first four semesters [16]. The musculoskeletal system is the superordinate topic in the first semester, followed by internal organs (metabolism, cardiovascular system, respiration, fluid and electrolyte balance, hormones) in the second semester, nervous and sensory systems in the third semester, and finally reproduction, blood and immune systems in the fourth semester. Working on patient cases together in the POL tutorials with six students, one medical tutor and one student co-tutor is useful for the acquisition of basic science and clinical knowledge and problem solving skills [19]. This method is considered both motivating [20] and supportive to establish interdisciplinary thinking [21], communication skills, independent sustained learning and understanding of ethical aspects of the healthcare system [22]. § 5 of the examination regulations of the UW/H for the model course of studies in human medicine [23] provides for summative equivalence examinations for the first section of the medical examination (preliminary medical exam, M1) according to § 41 para. 2 no. 3 of the Regulations for the Licensing of Physicians (ÄAppO) [https://www.gesetze-im- internet.de/_appro_2002/BJNR240500002.html] within the framework of a special regulation for model courses of studies. For this purpose, three written and two combined examinations are taken. The two combined examinations are Objective Structured Clinical Examinations (OSCE) [24], while the three written examinations comprise tasks in the free-text format MEQ at the end of the second semester (MEQ-1), third semester (MEQ-2) and fourth semester (MEQ-3). The choice of these examination formats for equivalence exams reflects the central significance of the POL learning format in accordance with the concept of constructive alignment [10], [17], [25]. To prepare for these summative exams, a formative exam is taken, i.e. the MEQ*. As known from informal discussions, many students do not specifically prepare for this, since the MEQ* is not part of the overall grade of the equivalence examination.

Therefore, the aim of this study was to clarify to what extent the formative examination MEQ* at the end of the 1st semester in the study program of human medicine at UW/H can serve as a predictor for the summative examination MEQ-1, which is part of the equivalence examination replacing the state examination at the end of the second semester. Here, the following potential influencing variables of the exam performance were considered: Age, gender, high school graduation grade, professional background, and self-efficacy expectancy. Self-efficacy from Bandura's social cognitive theory of human agency represents an essential motivational component, namely a person's internal personal belief that he/she can make substantial contributions [26], [27]. Accordingly, self-efficacy also plays an important role in learning and developing new competencies [28], especially in problem-based learning [29]. 

It can be assumed that, on the one hand, people with good problem-solving and learning strategies, mapped by their high school graduation grade [30], [31], [32] and their own self-efficacy expectations [26], [33] are more likely to achieve better results and, on the other hand, people with subject-specific prior knowledge, in the sense of previous professional knowledge and associated higher age [34], might also have an advantage. 

## Participants and methods

### Participants

We investigated students of human medicine at Witten/Herdecke University in the fall semester 2017/18 (cohort 45, N=44) and the spring semester 2018 (cohort 46, N=44). They were informed about the background of the study and provided their written consented. Data were used in anonymous form in accordance with the provisions of the Federal Data Protection Act (DSGVO) and the ethical standards of the Declaration of Helsinki [35]. The procedure was approved by the Ethics Committee of the UW/H (No. 39/2018). The personal data available in the electronic campus management system of the university (gender, age, high school graduation grade, and professional background in the healthcare sector, operationalized as professional training, previous studies in biology or biochemistry, number of internships, and/or a voluntary social year) were used with the consent of the students. 

#### Survey methodology

The tasks of the Modified Essay Questions (MEQ) comprise a case study with several related, sequential questions, which have to be answered in a structured way in short free texts [11]. As students work through the case, they receive new information on each page of the exam that might answer the questions on the previous page. Therefore, turning back pages is not allowed in the paper-pencil version of the MEQ [17], [36]. Edited answer sheets must be placed in a separate envelope so that they cannot be subsequently corrected. 

Medical students of the UW/H take the formative MEQ* at the end of the first semester under examination conditions. It is adapted to the level of knowledge and structured like a summative MEQ. The content of the formative MEQ* was the same in the fall semester 2017/18 and the spring semester 2018 and the scope was reduced to two patient cases and a few additional free questions. It consisted of two case histories on acute cholecystitis and traumatic shoulder dislocation with a total of 17 case-related questions on specific subjects and 5 free questions (#22 questions). A total of 116 points could be obtained. The students had 90 minutes to complete the test. Immediately afterwards, the exam was corrected within 75 minutes using the student peer review process based on an answer horizon provided by the Examination Office of the Dean of Students Office. Thus, students receive immediate feedback on their performance and can align their individual learning strategies on the assessment of the learning outcome in accordance with constructive alignment [2]. To verify the points assigned by the fellow students and the quality of this feedback, all students' answer sheets were again evaluated by a qualified author (OB) and the scores from the peer review process adjusted accordingly.

Due to an increased number of questions on the topics internal organs and musculoskeletal system, students had six hours to complete the summative MEQ-1. MEQ-1 for year 45 in the fall semester 2017/18 comprised five case histories with 31 case-related questions and 13 free-response questions (#44 questions), while year 46 in the spring semester 2018 comprised five case histories with 36 case-related questions and 9 free questions (#45 questions). Students' scores for each task in the summative MEQ-1 were provided by the UW/H Dean of Human Medicine's Office of Examinations. Overall, students were able to score a maximum of 235 (#45) or 255 (#46), respectively (see table 1 (Tab. 1)).

In the present study, students completed the General Self-Efficacy Expectation (SWE) scale according to Schwarzer and Jerusalem before starting the exam with the formative MEQ* to assess the influence of the students' own competence expectation to deal with difficult situations [37]. The 10 items of the four-point Likert scale with the same polarity were answered with the answer options (1) not true, (2) hardly true, (3) rather true, and (4) true exactly, and added up for the sum value. For example, one of the SWE items is “I always succeed in solving difficult problems when I try.” The SWE scale has good internal consistency in German samples, ranging from Cronbach's alpha=.80 to .90 [38]. Results on the validity are available from empirical studies showing theory-consistent positive correlations with dispositional optimism and job satisfaction, and close negative correlations with anxiety, depression, and burnout [33].

#### Statistical analyses

During the preliminary analyses, the sociodemographic variables age at baseline, gender and high school graduation grade, as well as the dependent variables total scores in the formative MEQ* and summative MEQ-1 for years 45 and 46, and self-efficacy expectancy were tested for normal distribution using the Kolmogorov-Smirnov test. As the assumption of normal distribution could not be confirmed, non-parametric testing was performed. For comparisons between the independent groups of students (year 45 vs 46), Mann-Whitney U tests were performed. Test size was analogously converted to Cohen's d effect size [39], [40]. Associations between variables were calculated by means of correlation analyses (Spearman rho) with correlation coefficient r as effect size. Multiple regression analyses were used to predict the score obtained on the MEQ-1 by the variables age, high school graduation grade, prior knowledge (occupation, studies), self-efficacy expectation, and MEQ* score. The significance level was set at p<.05 for these correlation and regression analyses performed with SPSS 26, and at p<.01 for the Mann-Whitney U tests after Bonferroni correction of the α-error [41].

## Results

### Description of the sample and group comparison

88 medical students from two cohorts of the UW/H (45 and 46) were included in this study. With regard to the variables age at beginning of the study, gender distribution, high school graduation grade as well as completed vocational training, pre-study, internships or voluntary social year and self-efficacy expectations, the two cohorts did not differ significantly and could therefore be considered as one sample for further analysis. 

On average, students (46 females, 42 males) were 22.3±2.5 years old (ranging from 19 to 29 years) at the beginning of the study. The high school graduation grade was 2.1±0.4 (ranging from 1.2 to 3.2), with females having a better high school graduation grade than males (1.96±0.42 versus 2.15±0.39; Mann-Whitney-U=700, p=.026, d=.488), which was not significant after α-correction. Prior to the start of the study, 36 students (40.9%) had completed a voluntary social year or learned a profession in the healthcare sector, e.g. general healthcare and nursing, physiotherapy or paramedic. Overall, 80% of the professions in this sample were in the medical field. 15 students (17%) had been engaged in a pre-study program, e.g., biology, nursing, or dentistry. In addition to the six-month nursing internship required to enter the study program, 67% (N=59) of all participants had completed other internships. The average self-efficacy expectancy of the students of 30.2±3.4 corresponds to the reference mean of 29 points [37].

#### Demographic characteristics and MEQ results 

Students scored a total of 66.9±13.9 (year 45 in the fall semester 2017/18) and 65.4±13.1 (year 46 in the spring semester 2018) on the formative MEQ* with a maximum score of 116. This small difference between the two cohorts is not significant. The points awarded by medical students in the peer-reviewed formative MEQ* are not significantly higher than in the objectified post-evaluation by a qualified author (OB) (see table 2 (Tab. 2)). Both total scores are highly correlated with each other (Spearman: r=.837, p=.000). The closest match is found in the subject anatomy (r=.933), followed by biochemistry (r=.904), physiology (r=.834), radiology (r=.775) and finally clinical reasoning (r=.463) (results of all correlation analyses p=.000). For further analyses, the results in the formative MEQ* as determined by the qualified author (OB) were used.

In the summative MEQ-1, year 45 achieved an average of 160.8±29.3 points out of 235 maximum possible points (corresponding to 68.4%) and year 46 achieved an average of 193.8±29.6 points out of 255 possible points (corresponding to 76%). There are no significant differences between the cohorts in the total points achieved after their z-transformation (U=909, p=.753, d=.105). 

The assessment of a potential relationship between age at the beginning of the study and MEQ results revealed that there is a moderate positive correlation (r=.306, p=.004) to the formative MEQ* and to the same extent one semester later to the summative MEQ-1 (r=.307, p=.004). In addition, there is a positive correlation between age at the beginning of the study as well as the high school graduation grade (r=.341, p=.001) which is due to the waiting semesters until the beginning of the study. There are no statistically significant gender-specific differences in the total score achieved on the MEQ* and MEQ-1. Moreover, high school graduation grades and self-efficacy expectations show no significant correlation to the score in the formative MEQ* or summative MEQ-1 (results of all correlation analyses p>.050).

Consideration of the variables related to students' professional experience did not reveal any significant differences for internships or a voluntary social year, respectively, with respect to the total score in the formative MEQ* and summative MEQ-1. However, in case of a completed pre-study (N=15), the points achieved in the formative MEQ* were higher than without pre-study (U=363, p=.021, d=.437). However, this difference is not significant, nor is it significant one semester later for scores on the summative MEQ-1. Students with a completed vocational training (N=36) scored higher on the MEQ* (U=685, p=.033, d=.466), as well as one semester later with significant difference on the MEQ-1 (U=615, p=.009, d=.607) (see table 3 (Tab. 3)). 

#### Predictors of MEQ results 

In multiple regression analyses, all influencing factors were considered together as potential predictors of scores achieved on the formative MEQ* and summative MEQ-1 (each as a dependent variable). Assessment of the predictors for the MEQ* revealed that the variables age, high school graduation grade, vocational training, and pre-study recorded during the admission process were significant predictors of performance on the MEQ*, while self-efficacy expectancy was not (see table 4 (Tab. 4)). However, this model explains only 23.1% of the variance, leaving other unknown variables to account for performance. The common variance components of the variables age, vocational training, and pre-study were differentiated in a stepwise regression analysis. In this analysis, age alone (β=1.74, T=3.23, p=.002) accounted for 10.8% of the variance.

The consideration of age, high school graduation grade, vocational training, pre-study, and self-efficacy expectations in the regression analysis revealed that these were not significant predictors of the total score on the MEQ-1. In contrast, the score from the formative MEQ* alone was able to resolve 40.5% of the variance (β=1.58, T=7.61, p=.000), and 44.4% in the joint model with the aforementioned variables (see table 5 (Tab. 5)). The significant correlation of positive proportionality between the formative MEQ* and the summative MEQ-1 in year 45 (r=.769, p=.001) and year 46 (r=.684, p=.001) is a proof of content validity [42]. 

For a differentiated analysis, the five medical subjects identified in both the formative MEQ* and the summative MEQ-1 (see table 1 (Tab. 1)) are considered as independent variables in the regression analyses. Scores in the subjects of anatomy, physiology, clinical reasoning, biochemistry, and radiology in the MEQ* explained 53.5% of the variance in the MEQ-1 (see table 6 (Tab. 6)). Except for radiology, all proved to be significant predictors. Results in anatomy alone explained 36.2% of the variance and together with clinical reasoning explained another 11.2% (47.4% total). In contrast, physiology and biochemistry only have a weak predictive effect. 

## Discussion

The purpose of this study was to determine whether a formative examination such as the MEQ* in the model course of study in human medicine at Witten/Herdecke University can serve as a predictor for the summative examination MEQ-1 one semester later. In addition, high school graduation grade, professional experience, self-efficacy expectancy, age, and gender were considered as possible influencing variables.

### Gender, high school graduation grade, and self-efficacy expectancy

Female students did not perform better than male students on both the MEQ* and MEQ-1, despite a gender-related difference in the high school graduation grade that narrowly failed to demonstrate statistical significance. This observation is similar to that among medical students at Heidelberg University [32]. The second state exam in fall 2018 also showed comparable exam performance for all medical students in Germany with female students achieving 79.0% versus male students achieving 79.2% of the total score [42]. In their meta-analysis on the prediction of academic success, Trapmann et al. [30] reported that in dental and veterinary medicine programs, the validity of the high school graduation grade was higher for pre-clinical than for clinical semesters. The same applies for human medicine with some 23% clarification of the variance in performance by previous academic performance at the beginning of the study and a total of about 9%, as demonstrated by Ferguson et al. [43] in a systematic literature review. Thus, previous academic experience is a good but not perfect predictor of performance in medical education. Moreover, gender-specific performance differences were rather small and only reach statistical significance in large cohorts. Thus, even in the present study, with moderate prediction for the formative MEQ*, there was no significant relationship between the high school graduation grade and performance in the formative MEQ* or in the summative MEQ-1. The same applies for the students' self-efficacy expectations [38], thus not confirming the findings of Klassen & Klassen [28] and especially Demirören et al. [29] on the effect in the context of problem-based learning. 

#### Age and previous professional training

The total score in the formative MEQ* shows a moderate positive correlation with age for year 46 (spring semester 2018), which is also positive for year 45 (fall semester 2017/18), but fails to reach statistical significance. The positive age effect is also still detectable one semester later for the MEQ-1. Older students had acquired professional experience in their waiting semesters through a pre-study, vocational training in the healthcare sector, specific internships or a voluntary social year (FSJ). However, internships and FSJ had no relevant influence on the results in the formative MEQ* or summative MEQ-1, as it seems that the acquired practical knowledge without a structuring theoretical foundation does not provide an advantage for the academic performance required in the exams. In contrast, students with pre-study (17% of the sample) performed better in the formative MEQ* in group comparison and achieved higher scores in physiology. Students with previous professional training (40.9% of the sample) also scored higher on the formative MEQ*, especially in the subject of anatomy. Medical students appear to benefit from both types of previous education: firstly from the structural, subject-independent previous education of a pre-study, in which independent university learning is learned, and secondly from the theoretical-practical previous education of a vocational training in the healthcare sector (80% of the sample) associated with content knowledge from vocational school and practical experience. However, this advantage seems to apply only until the end of the first semester for the formative MEQ*, not until the summative MEQ-1 that is taken one semester later. In terms of previous academic experience, this corresponds to the results of Ferguson et al. [43]. Parallels can also be found in a study by Grendel et al. [34] examining the effects of professional experience of vocationally qualified students. The duration and relevance of previous work experience seem to have a significant influence on the academic success.

#### Formative MEQ* and peer review process

The predictive significance of the formative MEQ* score for subsequent performance on the summative MEQ-1 was confirmed in the regression model with a 40.5% variance explanation. Moreover, correlation analyses showed a high content validity. Regarding the significance of specific subjects in the formative MEQ*, the particular importance of anatomy and clinical reasoning becomes clear. Traditionally, anatomy is the most challenging subject to learn at the beginning of medical studies, and this is also the case with the superordinate topic musculoskeletal system in the first semester of the model course in human medicine at Witten/Herdecke University. Clinical reasoning is trained by means of Problem-Oriented Learning (POL) and can be best tested using MEQ free-text questions according to constructive alignment [17], [18]. The points awarded in the formative MEQ* in peer review are not significantly higher as compared to those in the subsequent correction by the professionally qualified author (OB). Thus, despite the rather favorable evaluation by fellow students, student peer review seems to be an efficient method to provide quick feedback in a formative examination. The high correlation between peer review and objectified post-evaluation proves the reliability of the MEQ*, which is highest for anatomy and – as expected – lowest for clinical reasoning [14], [15], [44]. The problem, however, is that some students do not prepare for the formative exam. If the formative MEQ* had a pass mark of 60% [45], as is the case for the first state examination according to § 14 of the Regulations for the Licensing of Physicians (ÄAppO, 2002), more than half of the students would not pass. The average score achieved corresponds to 57% and is thus clearly above the average 30% score achieved by psychology students at the UW/H in the formative Progress Test Psychology [46], but demonstrates that students do not yet sufficiently realize the purpose and benefit of formative tests as feedback about their own level of knowledge, for learning motivation and reduction of test anxiety [47].

## Limitations

The small sample size of 88 medical students from two semesters should be taken into account. Moreover, they represent a heterogeneous group with regard to age and previous professional experience. In this respect, our findings can probably not be generalized, since they could be a matter of random, cohort-specific effects. Although the peer-reviewed results of the formative MEQ* were not significantly different in the post-correction by the professionally qualified author (OB), post-correction was difficult, as the students' responses did often not correspond to the response horizon. Thus, it was at the discretion of the post-correction staff how to score the answers given. The risk here is that different reviewers will arrive at different assessments of the answers which will reduce the reliability of the formative MEQ*. This is a fundamental problem of free text formats and requires a relatively high effort, both in the formulation of the response horizons and in the review process and correction [14], [15]. 

## Conclusions

The present investigation showed that there was a significant increase in knowledge during the second semester with year 45 (fall semester 2017/18) achieving an average score of 68% in the summative MEQ-1 and year 46 (spring semester 2018) 76%, respectively. The performance increase as compared to the result in the formative MEQ* is probably due to the systematic exam preparation, with approximately the same learning concept of POL tutorials during the first and second semester. The MEQ* as a formative test seems to give students feedback on their current knowledge level. Failure to pass could serve as a “wake-up call” for many students to intensify their learning efforts for the summative MEQ-1. Parallels can be found in Heeneman’s interview study [48], in which students confirm that they use the results analysis of the formative progress test to adjust their learning strategies. 

The fact students find out about the free text format in the formative MEQ* might also help them to adapt their learning style and exam preparation in accordance with the concept of constructive alignment. Thus, the goal of formative testing would be achieved by the MEQ* and the effort associated with the development and implementation of this type of formative testing would be justified for both lecturers and dean’s office at Witten/Herdecke University.

## Outlook

After it was demonstrated that the outcome of the formative MEQ* at the end of the first semester is a significant predictor for the outcome of the summative MEQ-1 at the end of the second semester, one relevant objective for further investigations would be to investigate for which period in the course of the study the MEQ* retains its function as a predictor. Does the performance in the formative MEQ* exam also say something about the overall summative equivalence exam (M1, preliminary medical exam)? It is quite interesting that – with respect to the subjects tested in the MEQ* – anatomy and clinical reasoning are paramount for the score in the summative MEQ-1. Since the performance in the subject of clinical reasoning seems to be unrelated to the overall scores of the other subjects, the teaching concept of problem-based learning has a particularly important role to play in this acquisition of competencies. From the low average score and the individual student statements it can be deduced that some students probably only prepare specifically for the formative MEQ*. In order to increase the benefit of this formative examination in its function as a useful feedback, a thorough exam preparation by of the students would be desirable. It would be important to investigate in further studies to what extent feedback from the formative MEQ* is actually used by the students to change their learning strategies.

## Competing interests

The authors declare that they have no competing interests. 

## Figures and Tables

**Table 1:**
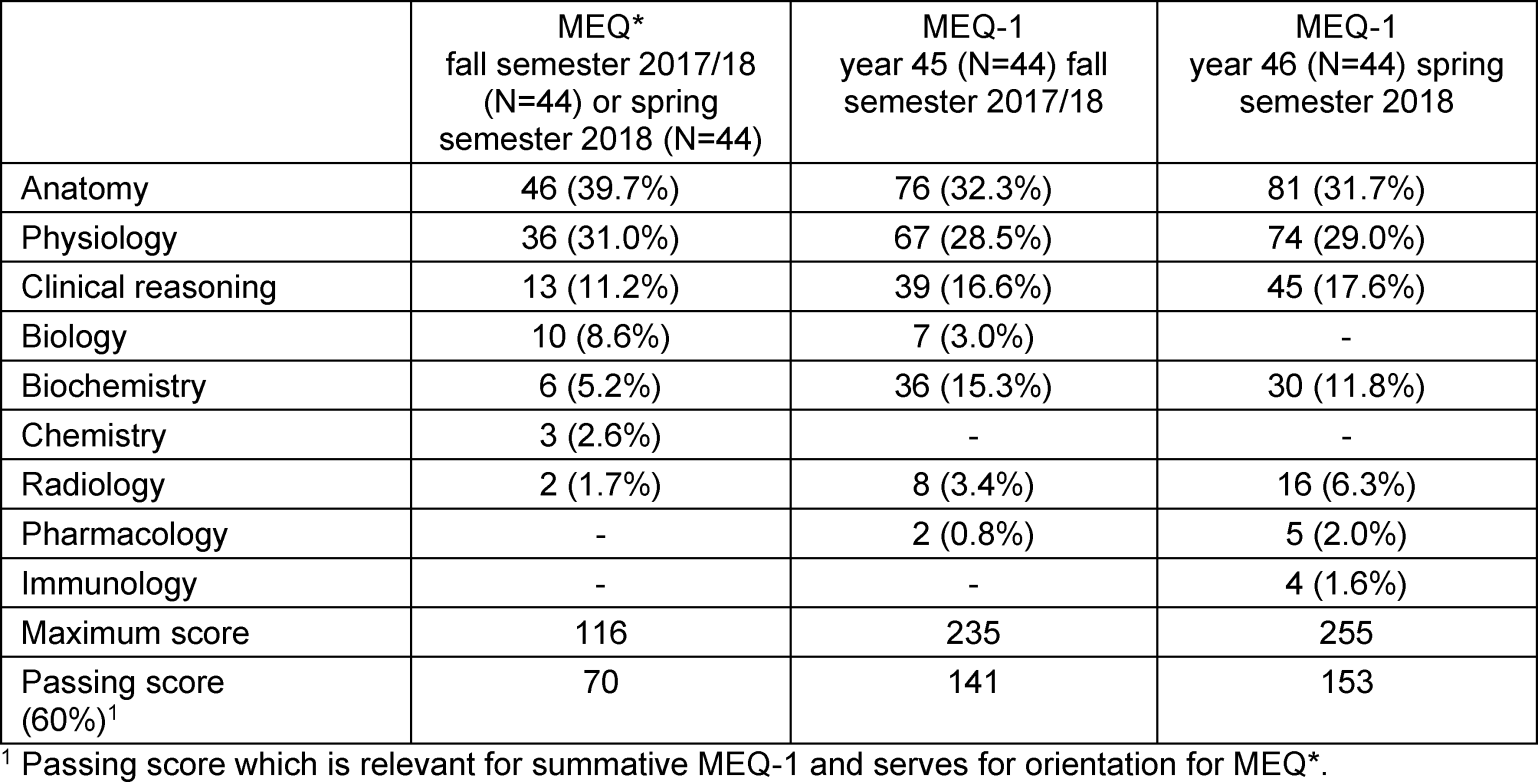
Distribution of the points that can be achieved in the formative MEQ* and summative MEQ-1 by subjects (score, percentage)

**Table 2:**
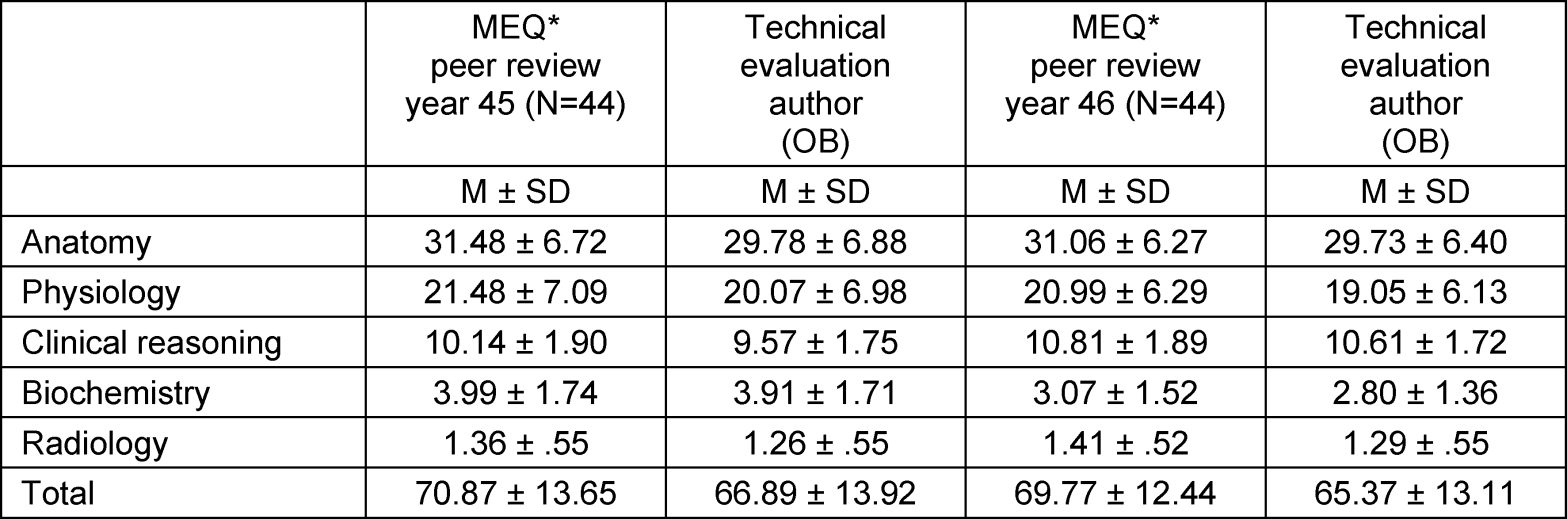
Comparison of results in formative MEQ* (mean, standard deviation) by peer review of medical students and professional evaluation by an author (OB)

**Table 3:**
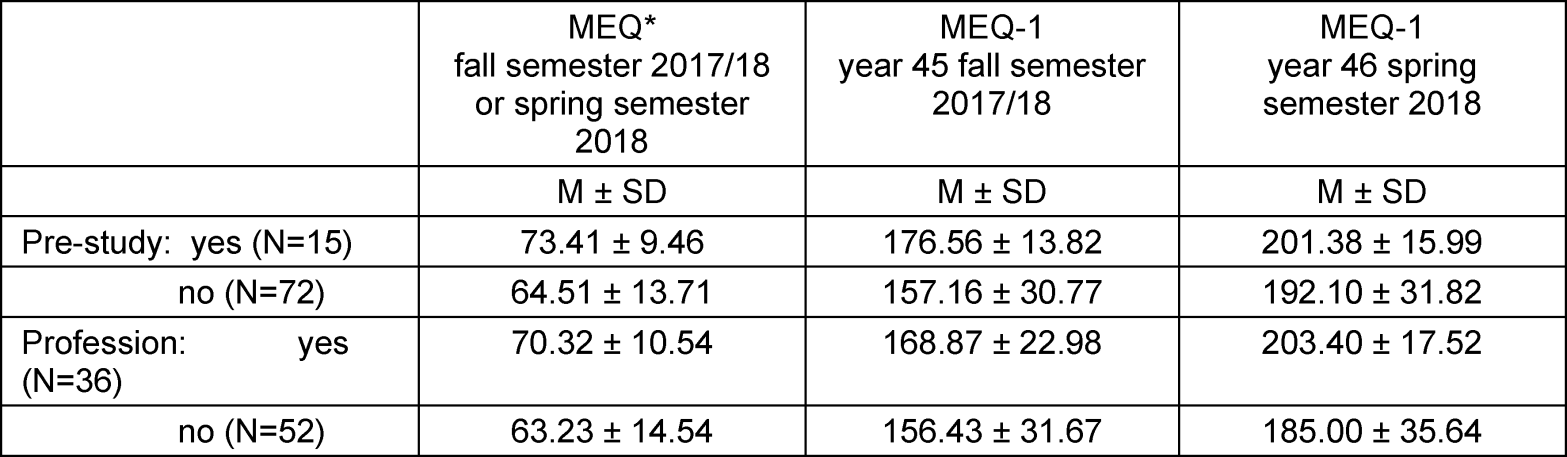
Group comparison on professional knowledge (undergraduate or professional) and results in formative MEQ* and summative MEQ-1 (mean, standard deviation)

**Table 4:**
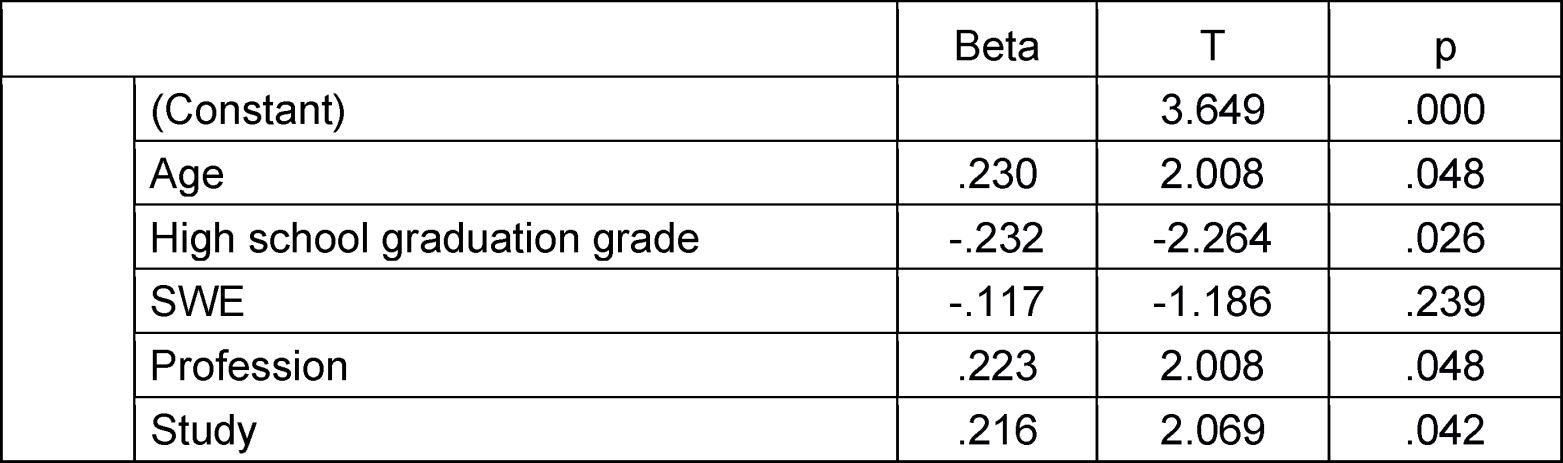
Predicting outcomes in the formative MEQ* (influencing variables in regression analysis: Age, high school graduation grade, self-efficacy expectancy, vocational training, and pre-study) (N=86) (regression coefficient beta, test value T, significance)

**Table 5:**
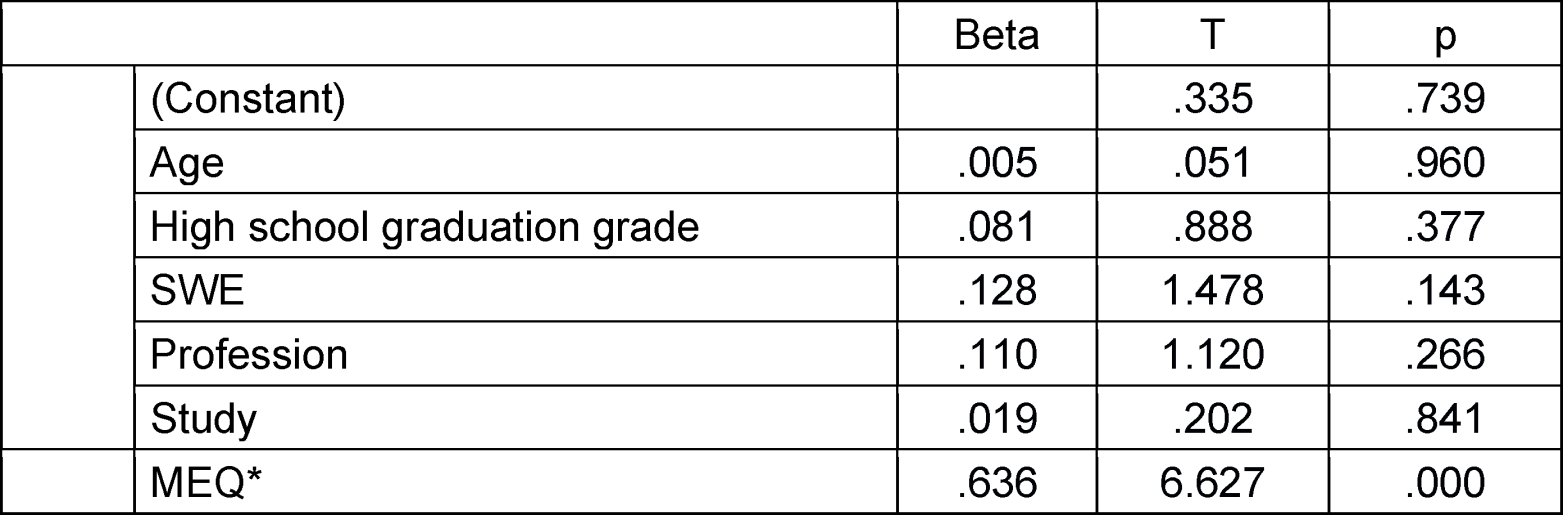
Predicting outcomes in the summative MEQ-1 (influencing variables in regression analysis: Age, high school graduation grade, self-efficacy expectancy, vocational training, pre-study and formative MEQ*) (N=88) (regression coefficient beta, test value T, significance)

**Table 6:**
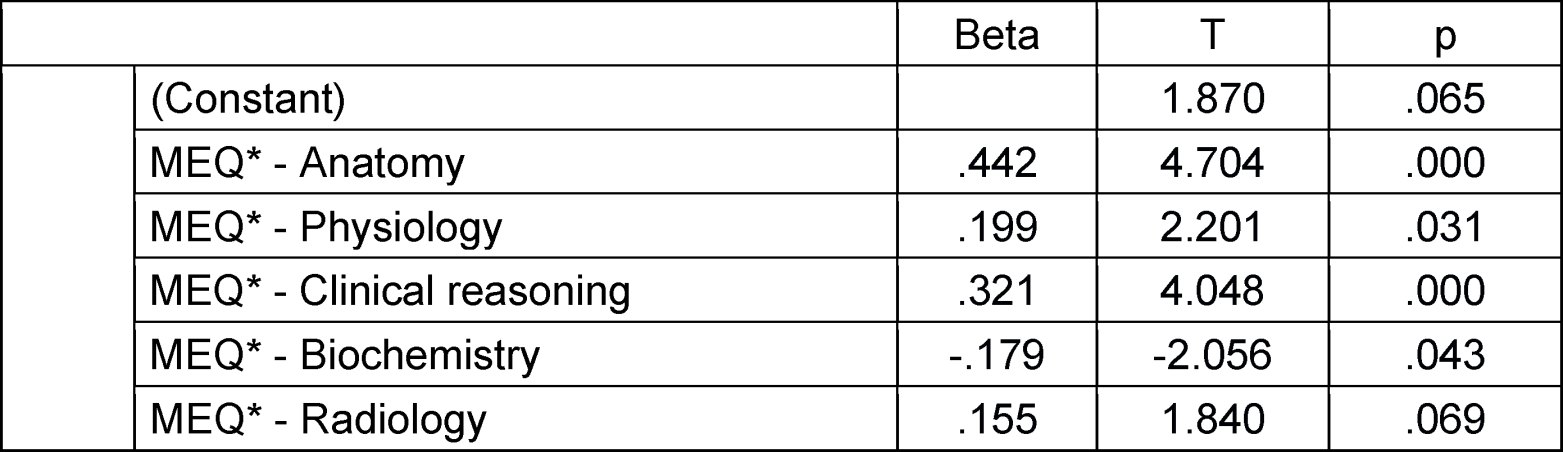
Predicting outcomes in the summative MEQ-1 (influencing variables in regression analysis: subjects of the formative MEQ*) (N=88) (regression coefficient beta, test value T, significance)
